# Perspectives on Genetic and Genomic Technologies in an Academic Medical Center: The Duke Experience

**DOI:** 10.3390/jpm5020067

**Published:** 2015-04-03

**Authors:** Sara Huston Katsanis, Mollie A. Minear, Allison Vorderstrasse, Nancy Yang, Jason W. Reeves, Tejinder Rakhra-Burris, Robert Cook-Deegan, Geoffrey S. Ginsburg, Leigh Ann Simmons

**Affiliations:** 1 Center for Applied Genomics and Precision Medicine, Duke University School of Medicine and Health System, Durham, NC 27708, USA; E-Mails: mollie.minear@duke.edu (M.A.M.); allison.vorderstrasse@duke.edu (A.V.); teji.rb@duke.edu (T.R.-B.); bob.cd@duke.edu (R.C.-D.); geoffrey.ginsburg@duke.edu (G.S.G.); leighann.simmons@duke.edu (L.A.S.); 2 Duke Science and Society, Duke University, Durham, NC 27708, USA; 3 Duke University School of Nursing, Durham, NC 27708, USA; 4 Icahn School of Medicine at Mount Sinai, New York, NY 10029, USA; E-Mail: nyyduke@gmail.com; 5 Sanofi, Cambridge, MA 08807, USA; E-Mail: jason.reeves@sanofi.com; 6 Sanford School of Public Policy, Duke University, Durham, NC 27708, USA

**Keywords:** personalized medicine, genetic tests, genomic tests, clinical implementation, pharmacogenetics, knowledge gaps, physician education, return of research results

## Abstract

In this age of personalized medicine, genetic and genomic testing is expected to become instrumental in health care delivery, but little is known about its actual implementation in clinical practice. Methods. We surveyed Duke faculty and healthcare providers to examine the extent of genetic and genomic testing adoption. We assessed providers’ use of genetic and genomic testing options and indications in clinical practice, providers’ awareness of pharmacogenetic applications, and providers’ opinions on returning research-generated genetic test results to participants. Most clinician respondents currently use family history routinely in their clinical practice, but only 18 percent of clinicians use pharmacogenetics. Only two respondents correctly identified the number of drug package inserts with pharmacogenetic indications. We also found strong support for the return of genetic research results to participants. Our results demonstrate that while Duke healthcare providers are enthusiastic about genomic technologies, use of genomic tools outside of research has been limited. Respondents favor return of research-based genetic results to participants, but clinicians lack knowledge about pharmacogenetic applications. We identified challenges faced by this institution when implementing genetic and genomic testing into patient care that should inform a policy and education agenda to improve provider support and clinician-researcher partnerships.

## 1. Introduction

Recent scientific discoveries and technological advances in precision and personalized medicine (PPM) promise improved health outcomes based on the predictive value of an individual’s unique clinical, social, behavioral, genetic, genomic, and environmental information [1,2,3,4]. Genomic and genetic tools have begun and will continue to influence clinical practice by allowing physicians to assess genetic load and predict individual disease development [5,6,7]. For example, a recent review of pharmacogenetics in cardiology indicated there is significant support for its use in drug categories including statins, antiplatelet medications, oral anticoagulants, beta-blockers, and angiotensin-converting enzyme inhibitors [8]. In oncology, pharmacogenetics is poised to become the standard of care in oncology, by enabling the prediction of appropriate treatment and clinical response, prevention of toxicities, and identification of drug resistance in a range of cancers, including breast, colorectal, gastrointestinal, lung, pancreatic, head and neck, and hematologic [9]. Likewise, studies have shown that human genetic variants influence responses to therapies such as antiretroviral therapies in HIV [10], including variable drug metabolism, drug disposition, and off-site drug targets.

While the research advances in PPM are evident, the integration of genomics-based PPM tools into clinical practice remains slow [11] and few studies have documented the uptake of personalized medicine tools in clinical practice [12,13]. Inconsistent interpretation of pharmacogenetic test results, scarce clinical guidelines for prescribing on the basis of test results, and limited clinical decision support systems for most drugs with genetic testing indications have contributed to the ongoing challenge of effectively utilizing pharmacogenetic tools in routine clinical care [14,15]. Additionally, although electronic health records have the potential to serve as the portal for genetic and genomic information, current systems do not have the capacity to store and analyze genetic data in ways that are clinically useful [12]. Financial issues further compound the practical challenges; economic models to date have not sufficiently established the cost-effectiveness of genetic testing to justify reimbursement [16]. Finally, the focus on personalized genomics in research has resulted in an onslaught of debate about the obligations and ethics of providing individualized genetic results to participants in both clinical and research sequencing practices and protocols [17,18,19,20,21,22,23,24,25,26]. While professional guidelines suggest returning results that are clinically actionable and life-threatening, clinicians and researchers struggle to define which genetic variants meet this threshold [27,28] and what providers may be obligated to disclose [22]. It is also unknown how many secondary or incidental findings may be present in a single exome or genome; some reports have only found a small number of these variants [29] while others have found larger numbers [30]. In summary, PPM practices that are highly efficient and effective in research may not always translate into clinical practice, particularly in non-academic centers.

Among other leading academic institutions, the Duke University Health System (DUHS) has contributed substantially to basic and clinical research in the field of PPM [4,31]. Studies have ranged from the development of targeted therapies to innovative care models designed to deliver customized care [32,33,34,35]. In the context of DUHS’ lengthy and diverse history with PPM, the purpose of this survey study was to explore perspectives on implementing PPM in DUHS (a large academic institution), with a specific focus on genetics and genomics. The goal of the survey was not to develop broadly generalizable knowledge about clinician use of PPM, but rather gain some pilot insights into perspectives at this particular institute with a strong PPM research program. The research questions guiding this investigation were: (a) What is the level of understanding of PPM in the institution?; (b) To what degree are clinicians utilizing PPM in their current practice?; and (c) How do providers feel about patient access to research-derived genetic testing information? Findings from this research may be used to guide institutions in best practices for deploying genetic and genomic technologies in clinical settings at other large academic institutions.

## 2. Materials and Methods

The Duke Center for Applied Genomics and Precision Medicine conducted this study and the study qualified as exempt by the DUMC Institutional Review Board. The 36-question mixed method survey was developed to collect data on the state of personalized medicine implementation at Duke. A pilot test of the survey instrument was conducted with faculty PPM experts and non-PPM experts of various disciplines and feedback was incorporated into the final instrument. We assessed: (a) providers’ use of genetic and genomic testing options and indications; (b) providers’ perceptions of patient interest in genetic and genomic testing; (c) respondents’ awareness of pharmacogenetic applications; and (d) respondents’ opinions on returning research-generated genetic test results. To capture as many Duke faculty and providers as possible, we used a combination of listservs and targeted department contacts to reach a broad population (see Table 1 for approach strategy). Some potential participants received an invitation from multiple avenues. All potential participants were emailed the invitation with a link to an online survey administered using Qualtrics software (Qualtrics, LLC, Provo, UT). The survey was limited to adults (18 and older). Of 3817 approached participants, 198 responded, 197 consented, and 166 completed the survey (response rate of 5%; survey completion rate of 84%). All questions included a “decline to respond” option. The questions (Table 2) collected data on (a) demographics; (b) perspectives on genomics and personalized medicine; (c) use of personalized medicine applications in the clinic; and (d) awareness of personalized medicine applications. Definitions were provided for “personalized medicine” and “pharmacogenetics” and examples were given for several genetics or genomics terminologies.

The survey data were analyzed through Qualtrics. The data were anonymized with respect to respondents’ identifying information, and participation was incentivized with a drawing for one of three iPad Minis. Analyses included examinations of whether responses varied based on (a) role (e.g., service on the DUHS IRB, clinicians *vs.* researchers), (b) clinical sub-specialties, and (c) years since highest degree was earned.

**Table 1:** Advertising strategy for survey participant recruitment.

Contact	Recipients	Reminders	Responses
Direct E-mail:			
School of Medicine	2946	1751	
			139
Listserv:			
Trinity College of Arts & Sciences	634		
Fuqua School of Business	102		
Law School	61		
School of Nursing	74		
			59
TOTAL	3817	1751	198

**Table 2:** Survey questions.

Question	
Q1	(Consent)
Q2	I am a member of (Duke affiliation names)
Q3	I consider my role to be (faculty, healthcare provider, student, staff, *etc.*)
Q4	Are you involved in the conduct of research activities?
Q5	Do you consider yourself to be a specialist provider? (PROVIDERS ONLY)
Q6	Select one of the following specialty categories that best describes you. (PROVIDERS ONLY)
Q7	What is your highest level of education completed?
Q8	How many years has it been since you completed your highest degree?
Q9	In what kind of research are you currently involved? (e.g., clinical research trials, basic research, translational research)
Q10	In the past five years, have you served on the Duke Institutional Review Board?
Q11–12	*Not reported here*
Q13	(Agree/Disagree statements regarding personalized medicine in research) (See Figure 3)
Q14	In your opinion, when should genetic results obtained through research be returned? (See Figure 3)
Q15	Are you an investigator on clinical trials involving personalized medicine?
Q16	*Not reported here*
Q17	Which of the following assessments do you routinely use in your clinical medicine? (PROVIDERS ONLY—See Figure 1)
Q18	Which of the following tests do you routinely order in your clinical practice? (PROVIDERS ONLY—See Figure 1)
Q19	Which of the following support tools do you routinely use in your clinical practice? (PROVIDERS ONLY—See Figure 1)
Q20–21	*Not reported here*
Q22	Do you use pharmacogenetic testing in your practice? (PROVIDERS ONLY—See Figure 1)
Q23	How many drugs are you aware of that have a pharmacogenetic indication in the drug package insert? (See Figure 2)
Q24	How frequently do you get questions from your patients about genomic tests? (PROVIDERS ONLY—See Figure 1)
Q25	How frequently do you get questions from your patients about pharmacogenetic tests? (PROVIDERS ONLY—See Figure 1)
Q26–36	*Not reported here*

## 3. Results

### 3.1. Survey Respondents Characteristics

*Roles*: A total of 63% of respondents self-identified as being faculty, and 41% self-identified as being a healthcare provider (Table 3); respondents could select all role(s) that applied to them. The majority of respondents (74%) indicated involvement in research activities, and 11% of all respondents had served on the Duke IRB within the last five years. *Experience*: Most (83%) of the healthcare providers indicated being a specialist provider, with a combined total of 27 different reported specialties. More than half (54%) of all respondents reported having a medical degree. The respondents’ years since highest degree ranged from recent graduates to 45 years of post-degree experience with a median of 15 years. *Research*: Of those involved in research, 53% reported involvement in clinical trials, and 17% reported investigator status on a clinical trial involving personalized medicine.

**Table 3:** Characteristics of respondents.

Category	Question	Responses	*N*	Percent
Total Respondents			198	
Consented			197	99%
Completed			166	84%
Affiliation	Q2 (*N =* 195)	Duke University Health System	66	34%
		Duke University Medical Center	142	73%
		Duke University and affiliates	24	12%
		Other	16	8%
Role	Q3 (*N =* 195)	Faculty	122	63%
		Healthcare provider	80	41%
		Administrator	21	11%
		Student/trainee	18	9%
		Staff	31	16%
		Other	3	2%
	Q5 (*N =* 78)	Provider specialist	65	83%
	Q10 (*N =* 195)	IRB service within last 5 years	21	11%
Education	Q7 (*N =* 195)	Medical degree (includes MD/PhD)	105	54%
		Doctorate degree	31	16%
		Masters or Advanced degree	14	7%
		Bachelors degree	19	10%
		Associates degree	10	5%
		High school	15	8%
Years Since Education	Q8 (*N =* 156)	0–5 years	18	12%
		6–10 years	36	23%
		11–20 years	46	30%
		21–30 years	37	24%
		More than 31 years	18	12%
Research Involvement	Q4 (*N =* 195)	Involved in research	144	74%
	Q9 (*N =* 144)	Clinical trials	76	53%
		Basic	44	31%
		Translational	52	36%
		Implementation	32	22%
		Outcomes	55	38%
		Epidemiological	27	19%
		Other	16	11%
	Q15 (*N =* 135)	Investigator on clinical trial involving personalized medicine	22	16%

### 3.2. Clinical Implementation of Genomic Tools

**Figure 1:**
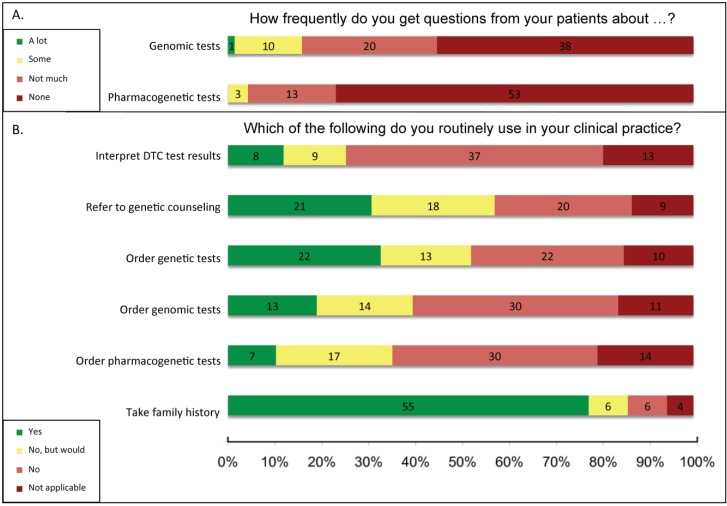
Clinical implementation of genomics tools. (**A**) In Q24 and Q25 (*N =* 69 for both), providers were asked how frequently they get questions from patients about genomic tests or about pharmacogenetic tests and were given a scale to indicate frequency: (a) “A lot,” (b) “Some,” (c) “Not much,” or (d) “None.”; (**B**) In Q18–19, providers were given a scale to indicate how routinely they used various genetic and genomic tools in their practice: (a) “Currently use,” (b) “Would use more frequently if patients requested,” (c) “Would use with timelier results,” (d) “Do not use but would like to implement,” (e) “Do not use,” (f) “Do not use and would not use,” (g) “Not applicable to my field.” Data from (b), (c), and (d) responses were combined into “No, but would” and data from (e) and (f) responses were combined into “No.” In Q19 providers were asked about “Interpretation of patients’ direct to consumer genomic test results” (*N =* 69) and “referral to genetic counseling” (*N =* 69), and in Q18 they were asked about pharmacogenetic tests (*N =* 70) and were provided with the examples of warfarin dosing and selection of antidepressants, genomic tests (*N =* 71) and were provided with the examples of genome sequencing and microarray profiling, and genetic tests (*N =* 70) and were provided with the examples of carrier status and diagnostics. For Q17, provider participants were given a similar scale to indicate their use of family history collection (*N =* 71): (a) “Currently use,” (b) “Would use more frequently if patients requested,” (c) “Do not use but would like to implement,” (d) “Do not use,” (e) “Do not use and would not use,” and (f) “Not applicable to my field.” Data from (b) and (c) responses were combined into “No, but would” and data from (d) and (e) responses were combined into “No.” Decline to respond selections were not included in the figure, but are included in the sample size given for each question. DTC, direct-to-consumer genetic test.

The 80 respondents who self-identified as healthcare providers were asked which genomics-based tools and tests they use routinely in clinical practice (examples of genomics-based tools were provided with the questions) (Figure 1). The majority of providers (55/72, 76%) reported that they collect family history; nearly one-third of providers reported referring to genetic counseling (21/69, 30%) or ordering genetic tests (22/70, 31%). However, few reported using genomic tests (13/71, 18%) or pharmacogenetic tests (12/69, 17%). Several respondents not currently using genetic, genomic, and pharmacogenetic tests reported that they would consider using them (19%, 20%, and 24%, respectively). The 12 providers that indicated they use pharmacogenetics in their practice (in Q22) also identified as researchers and 10/12 (83%) identified as specialists. The clinicians who also performed research (R) were slightly more likely to use genomics-based tools than clinicians who do not do research (NR) (Q18 interpret patients’ direct-to-consumer genomic test (DTC) results: R = 13% (7/54) *vs.* NR = 7% (1/15); refer to genetic counseling: R = 37% (20/54) *vs.* NR = 7% (1/15); order genetic tests: R = 38% (21/55) *vs.* NR = 7% (1/15); order genomic tests: R = 23% (13/56) *vs.* NR = 0% (0/15); order pharmacogenetic tests: R = 12% (6/55) *vs.* NR = 7% (1/15); and collect family history: R = 84% (48/57) *vs.* NR = 47% (7/15)). However, respondent numbers were insufficient to determine statistical significance. Very few respondents reported that their patients had asked questions about genomic or pharmacogenetic tests (Figure 1).

**Figure 2:**
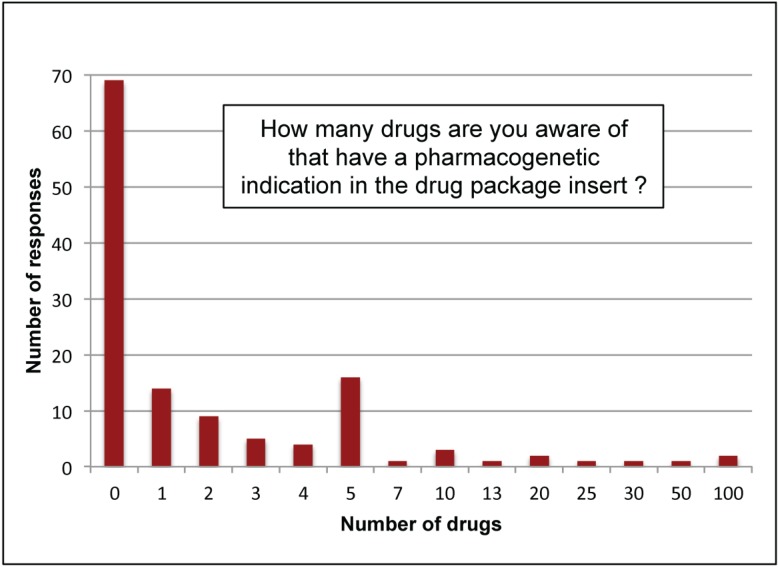
Awareness of pharmacogenetic indications. Respondents (*N =* 129) were asked how many drugs they were aware of with a pharmacogenetic indication in the labeling and were provided a blank field to enter a numeric answer. At the time of the survey, there were over 130 drugs reviewed by FDA with pharmacogenetic labeling information [36,37].

When all participants (not just providers) were asked how many drugs had a pharmacogenetic indication in the labeling, only two respondents (non-provider research faculty with > 15 years’ experience) indicated 100 drugs (Figure 2). In contrast, 91% (117/129) responded that they were aware of 5 or fewer drugs with a pharmacogenetic indication in their label and 53% (69/129) were not aware of any drugs with pharmacogenetic indications in labeling. At the time of the survey, as many as 130 drugs had pharmacogenetic information in the FDA-approved labeling [36,37] according to PharmGKB (www.PharmGKB.org).

### 3.3. Return of Research Results

**Figure 3:**
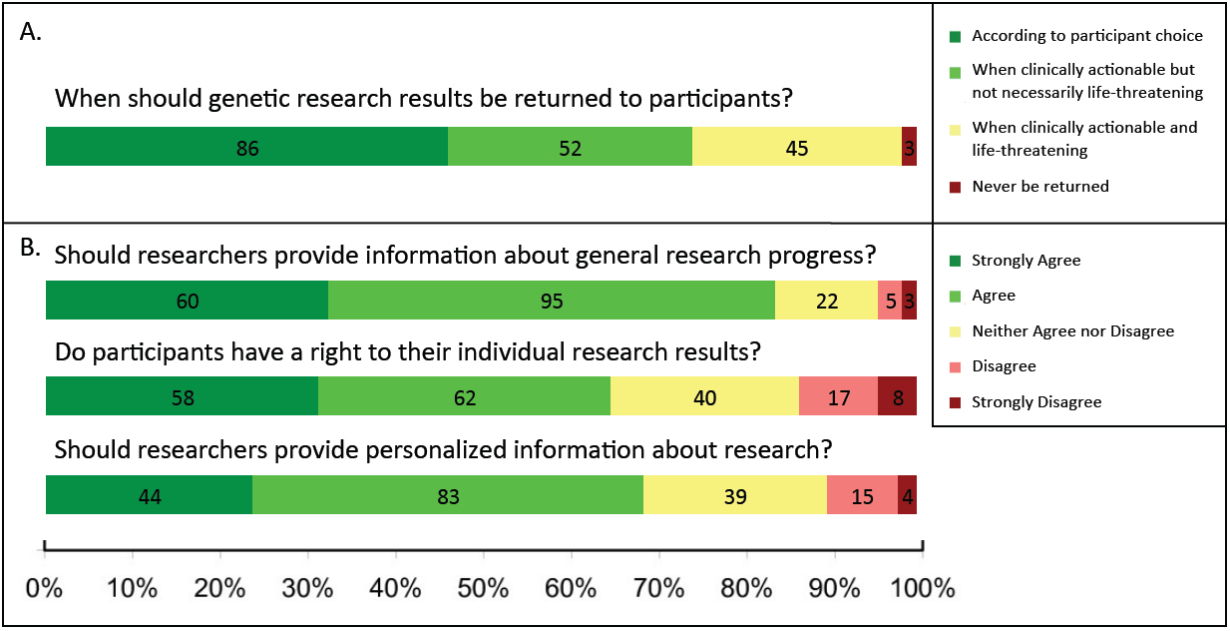
Attitudes towards return of research results to participants among researchers and non-researchers. (**A**) In Q14, all participants (*N =* 186) were asked: “In your opinion, when should genetic results obtained through research be returned?” and were asked to choose one of the following: (a) “According to participant choice,” (b) “When clinically actionable but not necessarily life-threatening (e.g., medication selection/dosing, family planning),” (c) “When clinically actionable and life-threatening,” and (d) “Never be returned.” Examples of genetic research were not provided to gain a general response to the term “genetic research.”; (**B**) In Q13, all participants (*N =* 186) were provided a 5-point Likert Scale (strongly agree-strongly disagree) to indicate their agreement with three statements: (1) “Researchers should make an effort to provide participants general information about the progress of the research,” (2) “Research participants have a right to their individual results generated from a research trial,” and (3) “Researchers should make an effort to provide participants personalized information about the research.” Decline to respond selections were not included in the figure, but are included in the sample size given for each question.

Respondents generally favored returning research results to participants (Figure 3), including both general progress about the research and individualized research results. The few (10%) respondents indicating that they disagreed or strongly disagreed with the notion of returning personalized research-based results to participants had been out of school longer (media *N =* 22.5 years since highest degree completed, standard deviation (SD) = 10.3) compared to respondents who strongly agreed, agreed, or were neutral toward returning results (16.8 years, SD = 10.8), a difference that was statistically significant (*p =* 0.036). Non-researchers were generally more supportive of returning results compared to researchers: all responding non-researchers were either supportive (46/50, 92%) or neutral (4/50, 8%) towards returning information related to the general progress of a research study back to participants; in contrast, 81% (109/135) of researchers reported that they would support returning this information (18/135, 13% neutral). Regarding personalized information about research, 80% (40/50) of non-researchers and 64% (87/135) of researchers supported returning this information to participants. Finally, 76% (38/50) of non-researchers and 61% (82/135) of researchers agreed that participants have a right to their individual research results. Other respondent characteristics had no obvious bearing on responses, including degree held, role, and IRB service.

## 4. Discussion

In this modest survey of faculty providers and researchers at DUHS (a large academic medical center), we found that few clinicians routinely implement genomic and genetic testing in their clinical practice but do use other PPM approaches like collecting family history and referral to genetic counseling. These findings are consistent with other studies and likely reflect several contributing factors [38,39,40,41]. First, participant knowledge about pharmacogenetic indications on drug labels in our sample was extremely limited; all clinician respondents underreported the number of drugs with current pharmacogenetic label indications. The highest number reported was 100 by two respondents who were both non-provider research faculty with >15 years’ experience. It is possible, if not likely, that respondents would not understand whether a “pharmacogenetic indication” in the label meant that the labeling required genetic testing or merely recommended such testing. Even so, since 91% responded that they were aware of 5 or fewer examples of pharmacogenetics in drug labeling and 53% were not aware of any pharmacogenetic indications in labeling this indicates a major education gap. Moreover, clinician-researchers reported higher usage of genomics-based tools in their clinical practice than clinicians who did not do research. This suggests the potential influence of participation in PPM research as a learning tool in the field. Second, tools such as family history and referrals were more likely to be a part of participants’ current medical work-up and treatment planning practices; these tools may not require as much additional knowledge/training to integrate. However, as electronic health records become capable of handling pharmacogenetic testing and interpretation [42], the integration of these tests into clinical care may increase [43]. Third, there was an educational gap in the integration of pharmacogenetic applications with newer providers more likely to utilize these tools compared to those who had completed their clinical training before pharmacogenetic testing became available [44]. This speaks to the need for not only provider education in the use of these tools through continuing medical education efforts, but also for support services to enhance integration [43,45]. Indeed, at least one other institution has successfully demonstrated that support strategies such as pharmacogenetics clinical services in a team-based approach can enhance the integration of pharmacogenetics into clinical care [46], and several programs in the U.S. and Europe are developing tools to support use of genomic tools [12,45,47]. Finally, few clinicians reported that patients asked them about genomic and pharmacogenetic tests, suggesting these providers may not see this as a necessary component of a clinical visit. Patients who undergo DTC testing will likely turn to their primary care provider for help interpreting their risk profiles; yet studies of DTC testing show that few providers feel prepared to answer questions about these tests [44,48], again suggesting the need for provider education and support services in this arena should DTC testing become more commonplace.

Our respondents were generally in favor of returning results back to research participants. Many respondents expressed support for returning research results according to participant choice; a majority of participants in a recent survey of genetics professionals expressed similar support [49]. This attitude is more liberal than the guidelines issued by the American College of Medical Genetics and Genomics, which state that clinically actionable and life-threatening data should be returned [17] but makes no mention of returning other data that participants may find important. However, researchers were somewhat less likely to report favoring the return of results than were non-researchers. This may reflect the fact that researchers feel less comfortable providing potentially clinically relevant information to a research participant because they do not have the skills or training to address questions or concerns that may arise, and supports the notion of strong clinician-researcher partnerships in both clinical and research endeavors in PPM. It may also reflect concerns about the additional workload that returning these results to participants would impose on researchers or about the current lack of infrastructure that would be needed to return these data. In contrast, other studies have shown that individuals believe researchers have an obligation to return individual genetic research results, but that they would also be content with limited results [50]. Research participants also have consistently expressed interest in having results from research into their genomes or exomes returned to them, in spite of some ambivalence and concern about receiving results regarding incurable conditions [51,52]. Older providers were less inclined to return research results than those who are younger. This may reflect a gap in knowledge and experience with genetic and genomic technologies, as well as limited participation in PPM research. Respondents were not asked for specifics on how research results should be returned (*i.e.*, with genetic counseling or clinical confirmation) [53].

The challenges of moving translational research into patient care in not unique to Duke, academic medical centers, or even the United States. Similar efforts to bridge the educational and awareness gaps in healthcare provider knowledge and use are found in Europe and across medical disciplines [12,13,41]. The results presented here are not necessarily representative of all medical centers or even academic medical centers, but reflect the difficulties of integrating PPM research into a healthcare system familiar with the scientific advances in PPM. What our study notes is a lack of utility even at an institute where we expected to find general awareness and acceptance of new technologies at the bedside. At another institute with less PPM experience, the resistance to uptake may be even greater than that found at Duke. However, this study has several limitations. First, the sample is not representative of the entire Duke University Health System and the majority of responding providers were specialists and not generalists. Our limited response rate and small sample size limited our ability to draw conclusions based on specialty. The response rate may reflect the targeted recruitment on participants engaged in PPM, whereby many eligible individuals who do not perceive PPM to be relevant to their specialty may have chosen not to participate. This targeted recruitment also may bias the data, possibly in favor of the use of PPM. Some genetic or genomic tools may be more applicable to some specialties (e.g., oncology) than others, and there also may be different issues with implementing PPM in primary care settings not reflected in these findings. Future studies with sufficient numbers to differentiate responses by specialty would be of interest. In addition, if the low response rate is a reflection of those who do not consider PPM as relevant to their disciplines, this could reflect a general apathy towards PPM and lack of knowledge of genomics applications or of the broader definition of PPM we used that does not limit PPM to genetic or genomics but additionally includes behavioral or environmental factors. Future studies should retain a broad sampling approach to gather diverse opinions, but could potentially enhance response rates by not excluding those who do not use or consider PPM in their current practice/research and improving the compensation for time in participating. Second, there is a likely selection bias among those who participated, and these findings may underreport the gaps in knowledge and utilization. Third, given that this study was conducted at an academic medical center, findings cannot be extended to other clinical settings like community hospitals or rural clinics and may not be applicable to other academic medical centers. Finally, as with any survey there are concerns about how well our survey instrument performed. Our survey included 36 questions and took participants an average of 10 minutes to complete; however, we still noted a 16% survey drop-off rate. In a target respondent pool that includes busy healthcare professionals, the amount of time they can devote to a survey is limited. Respondent satisficing, particularly with respect to taking shortcuts with responses to reduce participant effort and save time, may impact the quality of our data [54]. We did not include questions designed to evaluate our survey’s internal consistency reliability, but we did note discrepancies in the number of providers who reported ordering pharmacogenetic tests before being provided with a definition of pharmacogenetics *vs.* afterwards (7/70 before in Q18, 12/69 after in Q22).

## 5. Conclusions

Even at a large academic medical center with a strong focus on PPM, implementation of genomic and genetic technologies is limited. The findings highlight a need for ‘just-in-time’ information and provider support to enhance the integration of these PPM approaches into clinical care. Strong clinician-researcher partnerships likely provide the best means to enhance both clinical knowledge and utilization of PPM. While many clinicians and researchers support the return of research results according to participant choice, there is even greater support for the return of general research progress to participants; researchers should take this into account as they design study protocols. In addition to these partnerships, the institution must show a commitment to ongoing provider education and clinical support for implementation of PPM. This will need to include clinician education in multiple formats deemed preferable by providers, and ‘just-in-time’ clinical decision support via the electronic health record. This institution-wide survey provides a road map to guide policies at Duke and informs an educational agenda to strengthen PPM.
